# Quality and safety in residential aged care: an evaluation of a national quality indicator programme

**DOI:** 10.1111/imj.16052

**Published:** 2023-03-22

**Authors:** Maria C. Inacio, Tesfahun C. Eshetie, Gillian E. Caughey, Craig Whitehead, Johanna Westbrook, Len Gray, Peter Hibbert, Elizabeth Beattie, Jeffrey Braithwaite, Ian D. Cameron, Maria Crotty, Steve Wesselingh

**Affiliations:** ^1^ Registry of Senior Australians South Australian Health and Medical Research Institute Adelaide South Australia Australia; ^2^ UniSA Allied Health and Human Performance University of South Australia Adelaide South Australia Australia; ^3^ UniSA Clinical & Health Sciences University of South Australia Adelaide South Australia Australia; ^4^ Adelaide Medical School University of Adelaide Adelaide South Australia Australia; ^5^ College of Medicine and Public Health Flinders University Adelaide South Australia Australia; ^6^ Southern Adelaide Local Health Network SA Health Adelaide South Australia Australia; ^7^ Centre for Health Systems and Safety Research, Australian Institute of Health Innovation Macquarie University Sydney New South Wales Australia; ^8^ Centre for Health Services Research The University of Queensland Brisbane Queensland Australia; ^9^ Centre for Healthcare Resilience and Implementation Science, Australian Institute of Health Innovation Macquarie University Sydney New South Wales Australia; ^10^ School of Nursing, Faculty of Health Queensland University of Technology Brisbane Queensland Australia; ^11^ John Walsh Centre for Rehabilitation Research University of Sydney Sydney New South Wales Australia

**Keywords:** quality indicators, residential aged care, nursing homes, quality improvement, quality measurement

## Abstract

**Background:**

In Australia, 243 000 individuals live in approximately 2700 residential aged care facilities yearly. In 2019, a National Aged Care Mandatory Quality Indicator programme (QI programme) was implemented to monitor the quality and safety of care in facilities.

**Aim:**

To examine the validity of the QI programme indicators using explicit measure review criteria.

**Methods:**

The QI programme manual and reports were reviewed. A modified American College of Physicians Measure Review Criteria was employed to examine the QI programme's eight indicators. Five authors rated each indicator on importance, appropriateness, clinical evidence, specifications and feasibility using a nine‐point scale. A median score of 1–3 was considered to not meet criteria, 4–6 to meet some criteria and 7–9 to meet criteria.

**Results:**

All indicators, except polypharmacy, met criteria (median scores = 7–9) for importance, appropriateness and clinical evidence. Polypharmacy met some criteria for importance (median = 6, range 2–8), appropriateness (median = 5, range 2–8) and clinical evidence (median = 6, range 3–8). Pressure injury, physical restraints, significant unplanned weight loss, consecutive unplanned weight loss, falls and polypharmacy indicators met some criteria for specifications validity (all median scores = 5) and feasibility and applicability (median scores = 4 to 6). Antipsychotic use and falls resulting in major injury met some criteria for specifications (median = 6–7, range 4–8) and met criteria for feasibility and applicability (median = 7, range 4–8).

**Conclusions:**

Australia's National QI programme is a major stride towards a culture of quality promotion, improvement and transparency. Measures' specifications, feasibility and applicability could be improved to ensure the programme delivers on its intended purposes.

## Introduction

Population‐based health surveillance measures and an understanding of unwarranted variation are required to evaluate health systems and support evidence‐based quality improvement initiatives.^1^, ^2^, ^3^, ^4^ Quality and safety monitoring in the aged care sector have long been recognised as being of significant value for its vulnerable population.^5^, ^6^, ^7^, ^8^ The development and use of indicators to support quality and safety monitoring, identify unwarranted service variation, facilitate benchmarking and underpin quality improvement initiatives has been ongoing internationally for decades. For example, countries, such as the United States, United Kingdom, Sweden and Canada have comprehensive, mandatory and, in some instances, public, quality and safety reporting systems originating from the 1990s.^5^, ^8^ While existing Quality Indicator programmes (QI programmes) have shown varying success in regard to increasing performance transparency, promoting higher standards of care within provider organisations (e.g. ratio of skilled workers to residents) and informing practices (e.g. quality use of medicines), important limitations have been recognised, such as choice of data sources for monitoring and limited capture in some programmes.^5^, ^6^, ^7^, ^8^

In Australia, over 1.5 million individuals currently receive government‐subsidised aged care, at a cost of AU$23.6 billion yearly.^9^ While in 2006 a voluntary state‐based QI programme was established by the Victorian Public Sector Residential Aged Care Services,^10^ it was not until July 2019 that the Aged Care Quality and Safety Commission, the Australian government regulatory body, implemented the National Aged Care Mandatory QI programme to monitor the safety and quality of care provided to more than 243 000 annual residents of approximately 2700 residential aged care facilities. This original national QI programme objective was to ensure ‘providers will have robust, valid data to support continuous quality improvement in the care they provide to aged care recipients’.^11^ The programme's second objective of ‘over time, to give consumers transparent information about quality in aged care to assist decision making’ was added to the second version of the QI programme for implementation from July 2021.^12^ For the first 2 years (July 2019–June 2021), the QI programme required facilities to monitor and report on three domains: pressure injuries, unintended weight loss and use of physical restraints.^12^ In the QI programme's third year, starting July 2021, two indicator domains were added: falls and medication management.^12^ Further advancements in Australia's QI programme were announced by the Australian Government in May 2021 in response to the Final Report of Australia's Royal Commission into Aged Care Quality and Safety and were released late in 2022.^13^

We examined the validity of measures included in version 2.0 of the QI programme^12^ using validated explicit criteria to assess each indicator's importance, appropriateness, clinical evidence base, specifications, feasibility and applicability.^14^ This evaluation aimed to provide valuable insights into the ability of the current QI programme^12^ and future efforts based on these QIs, to support the monitoring of the quality and safety of care provided to Australia's residential aged care recipients.

## Methods

The Aged Care Quality and Safety Commission National Aged Care Mandatory QI programme Manual 2.0 Part A^12^ and quarterly reports (from 1 July 2019 to 30 June 2021) of the QI programme were reviewed.^15^ This evaluation focused on the five domains with eight indicators of the QI programme: pressure injuries, use of physical restraints, unplanned weight loss (significant and consecutive unplanned weight loss), falls and major injury (one or more falls and falls resulting in one or more major injuries) and medication management (polypharmacy and antipsychotic use).^12^ A modified version of the American College of Physicians (ACP) Measure Review Criteria was employed, which was developed by the ACP Performance Measurement Committee to review the validity of performance measures as indicators of quality of care included in the Medicare merit‐based incentive payment system and quality payment programme in the United States.^14^ We selected this tool because it has been validated, was purposely developed to evaluate the validity of quality metrics by members of an expert panel and has been employed in influential studies.^14^, ^16^ Our modification of the tools, which we do not expect to influence their properties, included changing the wording referencing ‘physicians’ to ‘providers’.

Using the modified version of the ACP Measure Review Criteria, we examined the performance of the indicators against the following criteria:

1. Importance
  - Meaningful impact: implementation of this measure will lead to a measurable and meaningful impact.
  - High impact: measure addresses a condition that has a high impact (high prevalence, high morbidity or mortality, high severity of illness and major individual or societal consequences).
  - Performance gap: current performance does not meet best practices and there is opportunity for improvement.
2. Appropriate care
  - Overuse: measure will promote stopping the use of a test or treatment in individuals in whom the potential harms outweigh the potential benefits.
  - Underuse: measure will encourage use of a test or treatment in individuals in whom the potential benefits outweigh the potential harms.
  - Time interval: time interval to measure the intervention is evidence‐based.
3. Clinical evidence base
  - Source: evidence forming the basis of the measure is clearly defined with appropriate references.
  - Evidence: evidence is high quality, high quantity and consistent and represents current clinical knowledge.
4. Measure specifications
  - Clarity: numerator and denominator are clearly defined. This includes outcome measures; numerators detail an outcome that is meaningful to the resident and under the influence of the providers' care. Denominator includes well‐specified and clinically appropriate exceptions to eligibility for the measure.
  - Clarity: all components necessary to implement the measure are clearly defined.
  - Validity: the measure correctly assesses what it is designed to measure, adequately distinguishing between good and poor quality.
  - Reliability: the measure is repeatable and precise, including when data are extracted by different people.
  - Risk adjustment: risk adjustment is adequately specified for outcome measures.
5. Measure feasibility and applicability
  - Attribution: level of attribution specified in measures is appropriate (measure ties the outcomes to the appropriate unit of the analysis) and is clearly stated.
  - Providers control: the performance measure addresses an intervention that is under the influence of the providers being assessed.
  - Usability: the results of the measure provide information that will help the provider improve care.
  - Burden: data collection is feasible and the burden is acceptable (low, moderate or high).

Each indicator's importance, appropriateness, clinical evidence, specifications, feasibility and rationale were assessed and rated by five of the authors (MI, GC, JW, PH and IC), who are researchers and clinicians with significant expertise in the area of quality and safety monitoring and evaluation. The reviewers were a convenience sample of individuals with expertise in quality and safety measurement (MI, GC, JW and PH), population health surveillance systems (MI and GC), geriatric and rehabilitation medicine (IC), pharmacoepidemiology (GC and JW), patient safety and evidence‐based care and evaluation (JW and PH). The reviewers were provided with documentation on the ACP criteria, instructions on how to score and complete a scoring sheet to collect the required information and supporting information about the QI programme for their reference (e.g. published reports, summary reports and manuals). The authors' rationales for scoring were also recorded. For each of the criteria, ratings ranged from whether the indicator does not meet the criteria (1 is the lowest rating) to whether it meets the criteria perfectly (9 is the highest rating). A median score of 1–3 was considered to not meet criteria, a score of 4–6 was considered to meet some criteria and a score of 7–9 was considered to meet criteria.^14^ The QI programme reports were summarised using descriptive statistics (see Appendix 1).

## Results

Table 1 provides a summary of the reviewers' assessment of the eight indicators included in the QI programme (see Appendix 2 for the reviewers' rationale and comments). All indicators, except polypharmacy, met criteria (median scores between 7 and 9) for importance, appropriateness and clinical evidence to support their inclusion in the QI programme. Polypharmacy met some criteria for importance (median = 6, range 2–8), appropriateness (median = 5, range 2–8) and clinical evidence (median = 6, range 3–8).

**Table 1:** National aged care mandatory quality indicator programme measure ratings

	Brief description†	Criteria score, median (min–max)				
Indicator		Importance	Appropriate care	Clinical evidence	Specifications	Feasibility and applicability
Pressure injuries	Percentage of residents with pressure injuries, reported against six pressure injury stages	9 (8–9)	7 (7–9)	9 (8–9)	5 (4–7)	5 (4–7)
Physical restraints	Percentage of residents who were physically restrained	9 (8–9)	8 (7–8)	7 (6–8)	5 (4–7)	6 (3–8)
Unplanned weight loss – significant	Percentage of residents with significant (5% of more) weight loss	7 (7–9)	7 (6–7)	7 (6–8)	5 (4–6)	6 (4–7)
Unplanned weight loss – consecutive	Percentage of residents who experienced consecutive weight loss – amount every month over three consecutive months of the quarter	8 (7–9)	7 (6–7)	7 (6–8)	5 (4–7)	6 (4–7)
Falls – one or more falls	Percentage of residents who experienced one or more falls	7 (6–8)	7 (6–7)	8 (7–8)	5 (3–7)	6 (4–7)
Falls – resulting in one or more major injuries	Percentage of residents who experience one or more falls resulting in major injury (defined as fractures, dislocations, closed head injuries with altered consciousness and/or subdural haematoma)	8 (7–8)	7 (7–8)	8 (7–8)	7 (4–8)	7 (4–8)
Medication management – polypharmacy	Percentage of residents prescribed nine or more medications (not including topical, dietary supplements, short term or PRN medications)	6 (2–8)	5 (2–8)	6 (3–8)	5 (2–8)	4 (2–8)
Medication management – antipsychotics	Percentage of residents who received antipsychotic medications	8 (7–9)	8 (6–8)	8 (7–9)	6 (4–8)	7 (4–8)

† All indicators are reported quarterly (90 days).

The indicators for pressure injury, physical restraints, significant unplanned weight loss, consecutive unplanned weight loss, one or more falls and polypharmacy met some criteria for validity of specifications (median scores = 5 for all) and feasibility and applicability (median scores between 4 and 6). Falls resulting in major injuries met criteria for specifications (median = 7, range 4–8) and feasibility (median = 7, range 4–8). Antipsychotic use met some criteria for specifications (median = 6, range 4–8) and met criteria for feasibility and applicability (median = 7, range 4–8).

## Discussion

Using a framework to examine the validity of the indicators included in the Australian National QI programme, we determined that the importance, appropriateness and clinical evidence base for the indicators were deemed high, except for polypharmacy. However, validity concerns regarding the feasibility and applicability of most indicators were identified.

We agree that most of the measures included in the Australian National QI programme are *important*, as they capture events with high impact on the aged care resident population. These measures are highly prevalent (e.g. physical restraint use, falls),^17^, ^18^ or cause high morbidity or mortality (e.g. pressure injury),^19^ or have high resident consequences (all measures except polypharmacy)^17^, ^20^, ^21^ or meet all of these criteria (e.g. unintended weight loss).^21^ These measures can influence improvements in residents' clinical outcomes, as demonstrated by their inclusion in international quality improvement systems.^5^ In addition, a gap in performance (e.g. national variation) in pressure injuries, physical restraints, unintended weight loss and antipsychotic use (e.g. variation in facility performance) has been reported nationally^22^, ^23^, ^24^ and it is likely that national differences in falls and polypharmacy also exist.^23^, ^25^ We agree that all of the measures except polypharmacy can inform changes in the *appropriateness of care*.^26^ For example, it is likely that measuring pressure injury incidence can inform quality improvement strategies and influence processes, treatments or activities to improve the likelihood of pressure injuries occurring. There was clear agreement that physical restraints and use of antipsychotics should be minimised and monitored to prevent overuse. However, clarification regarding family‐requested restraints (e.g. bed rails) and other types of restraints or options attempted is required. Seven measures were based on high‐quality *clinical evidence*, although certain components of the measures are complex (e.g. the definitions of ‘significant’ and ‘consecutive’ weight loss) and, as currently written in the QI programme manual, require refinement. In our evaluation, the polypharmacy measure only met some criteria for a *clinical evidence base*, as it provided limited insights into quality and safety of care without the context of residents' multimorbidity. Reviewers suggested that the numerical definition of polypharmacy does not discriminate between appropriate and inappropriate medication use, and, therefore, this measure might not be able to identify variations in appropriate care and not be able to influence practice changes. We suggest that a more appropriate focus is the quality of medication management rather than the number of medications.

The endorsement of quality indicators typically requires that the measure demonstrate importance, reliability and validity before it is deemed a candidate for endorsement. We agree that all of the measures' *specifications* in the QI programme except for falls resulting in major injuries had significant limitations. We believe that the current specifications for these measures limit their validity and ability to inform quality improvement activities or contribute to benchmarking amongst facilities. For example, the exclusion of individuals who do not consent to being assessed for pressure injuries and weight loss creates a risk for selection bias in resident inclusion. There are inappropriate denominators for some of the measures, for example, the denominator for the antipsychotic quality indicator. Antipsychotic use reporting should be restricted by residents' dementia status, who are those at most risk for inappropriate antipsychotic medication use, or at least be reported by those with and without dementia. Information regarding the reliability of the quality indicators is also another limitation of the current specifications. For example, there are several interrater reliability issues of pressure injuries classification, which requires clinical knowledge.^27^ In addition, the reliability of the measures is not reported. Finally, one of the most important features of indicators used for benchmarking is adequate risk adjustment, to ensure that comparisons between facilities are fair. Currently, none of the indicators in the QI programme are risk adjusted, resulting in an inability to compare care between facilities, or states, or other comparison groups. We have shown in previous work that age, sex and a diagnosis of dementia alone can significantly affect facility performance in quality indicators measured nationally,^23^ as others have shown internationally.^5^

We agree that all measures except falls met some criteria regarding *feasibility* and *applicability*. The measures employ the appropriate unit of analysis, for example, the rates/occupied bed days that account for the time individuals are ‘at risk’ for experiencing the events. In the second version of the programme, to improve audience (i.e. providers and public) interpretation, the proportion of residents who experienced the indicators is also reported.^24^ For providers to improve performance in the areas captured by these measures, they must be able to influence them. Currently, it is unclear whether that is possible for some of the measures. For example, physical restraints requested by family or polypharmacy may also be outside of their control. Another important feature of these measures is how usable they are for providers. At this time, it is likely that monitoring these indicators, if not already performed by the facility, could stimulate a review of facility activities that promote quality improvement. Finally, significant burden by the QI programme is put on the providers, to collect this information. Given the programme's reliance on manual data collection, the known challenges encountered by the sector with high staff turnover, and the need for significant staff training for the collection, the burden is high.^28^ Opportunities to reduce provider response burden exist and include leveraging providers' care management software or using existing national‐ and state‐level data collections to replace some of the manual data collection.^23^, ^25^, ^29^

Our assessment is limited by the small number of reviewers^5^ and the perspectives of the individuals involved in the review, mostly academics and clinicians. We did not involve residents, carers and those involved in data collection in residential aged care facilities in the expert panel. In addition, we focused on the indicators and the manuals developed to implement them and have not commented on other important aspects of the programme, including reporting, domains of quality care represented, benchmarking strategies and the identification and support of residential aged care facilities. We recognise that the second version of the National QI programme significantly improved the denominators' specifications for QIs; however, there was no progress on risk adjustment for the indicators in the QI programme, which is one of the more significant concerns.

Although our findings suggest that most of the indicators included in the current QI programme were important, can inform changes in the appropriateness of care and were based on appropriate clinical evidence, there remain areas in need of improvement, from measure specifications to data collection strategies, which cause significant provider burden. This study also highlighted the need for risk adjustment of the indicators in the current QI programme for benchmarking comparisons amongst residential aged care facilities. Future research is required to assess the domains of quality care included in the QI programme.

## Conclusions

The establishment of a national QI programme in Australia is a major stride towards a culture of quality promotion, improvement and transparency. Since 2019, this programme has established an infrastructure to measure, evaluate and report on important domains of quality of care. As the programme evolves, the specifications of the measures and feasibility and applicability, as identified in this evaluation, must be improved to ensure that the programme delivers on its intended purposes and benefits providers, regulators and above all the residents of these facilities.

## Supporting information


**Appendix 1.** Summary of the National Aged Care Mandatory Quality Indicator Programme Quarterly Reports

**Appendix 2.** National Aged Care Mandatory Quality Indicator Programme Measure Review, Reviewers' Rationale and Comments
