# Supplementary material for: Quality and safety in residential aged care: an evaluation of a national quality indicator programme
Source: Intern Med J. 2023 Mar 22;53(11):2073–8. doi: 10.1111/imj.16052 (PMC10946472; doi:10.1111/imj.16052)
Supplement: Supplementary file 1 — Appendix 1. Summary of the National Aged Care Mandatory Quality Indicator Programme Quarterly Reports [file IMJ-53-2073-s002.docx]

**Appendix 1. Summary of the National Aged Care Mandatory Quality Indicator Programme Quarterly Reports**

*Australian QI Program reports (See* ***Figure 1*** *for summary measures).*

The first published QI Program report (July-September 2019) captured information from 2413 facilities (94.5% of the 2688 expected) and the 8^th^ report (April-June 2021) from 2613 facilities (96.4% of the 2710 expected).

Between the July-September 2019 and Apr-June 2021 reports the crude published rates of pressure injuries/1000 care days decreased 13.1% (0.84/1000 to 0.73/1000 care days), with the largest decrease in stage 4 pressure injuries (50% decrease). The crude rates of intent to restrain decreased 18.7% (1.55/1000 to 1.26/1000 care days) and the rates of physical restraints device use decreased 24.9% (3.89/1000 to 2.92/1000 care days). The crude rates of significant unintended weight loss increased 5.4% (0.74/1000 to 0.78/1000 care days) while the crude rate of consecutive weight loss decreased 2.6% (0.77/1000 to 0.75/1000 care days).

**Figure 1. National Aged Care Mandatory Quality Indicator Program 1 July 2019- 30 September 2019 (Report 1) to 1 April 2021-30 June 2021 (Report 8), Indicator Crude N/1000 Care Days***


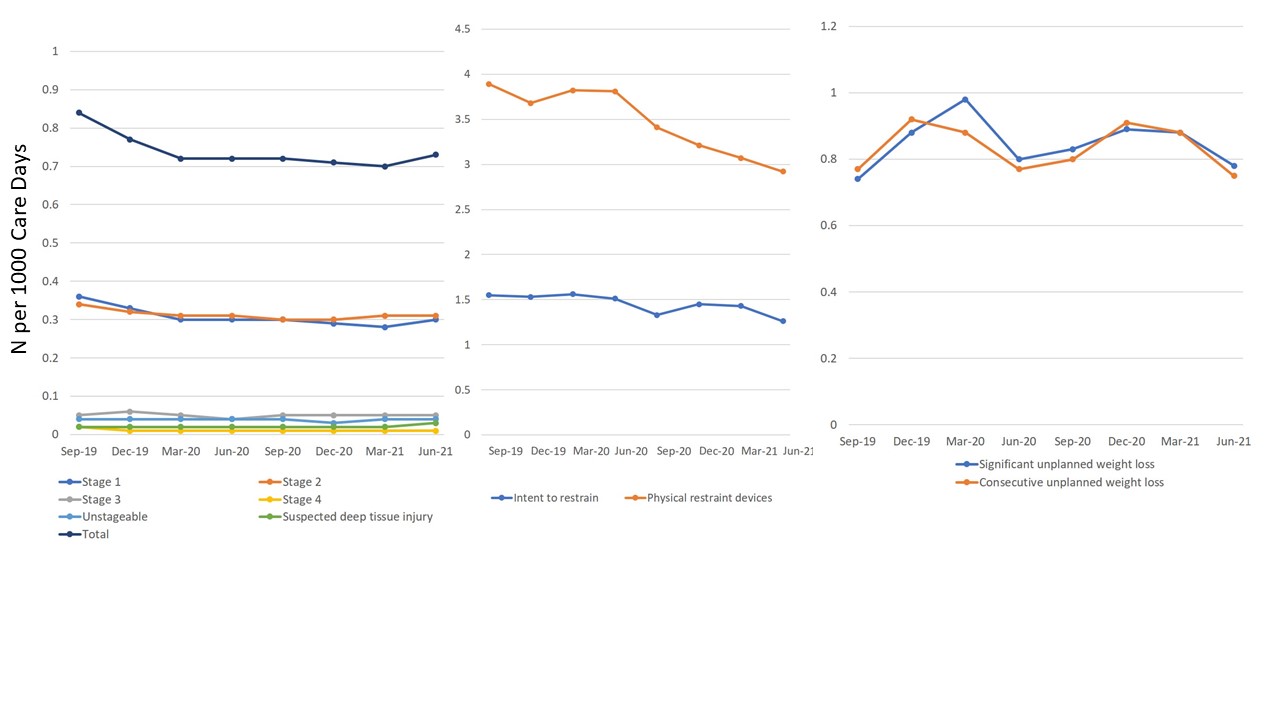


*Until 30 June 2021, the QI Program included 3 quality indicator domains: pressure injuries, physical restraint and unplanned weight loss. Since 1 July 2021, 2 additional domains (falls and major injury, and medication management) were included in the QI Program. Accordingly, the summary statistics presented in **Figure 1** only shows data for the original QI Program’s quality indicators: pressure injuries, physical restraint (intent to restrain and physical restraint devices), unplanned weight loss (significant and consecutive unplanned weight loss). From 1 July 2021, the QI Program requires residential aged care services to report on 8 quality indicators (5 domains): pressure injuries, physical restraint, unplanned weight loss - significant, unplanned weight loss - consecutive, falls-one or more falls, falls- resulting in one or more major injuries, medication management – polypharmacy and medication management – antipsychotics.
