# Supplementary material for: Quality and safety in residential aged care: an evaluation of a national quality indicator programme
Source: Intern Med J. 2023 Mar 22;53(11):2073–8. doi: 10.1111/imj.16052 (PMC10946472; doi:10.1111/imj.16052)
Supplement: Supplementary file 2 — Appendix 2. National Aged Care Mandatory Quality Indicator Programme Measure Review, Reviewers' Rationale and Comments [file IMJ-53-2073-s001.docx]

**Appendix 2. National Aged Care Mandatory Quality Indicator Programme-Measure Review, Reviewers’ Rationale and Comments.**

| **Indicator** | **Rationale / Comments from Reviewers** |
| --- | --- |
| **Pressure injuries** | **Importance**: High impact event with high morbidity and major individual consequences.^1-4^ Aligns with the principles of high-quality wound care.^5^ Some evidence that national performance gap exists.^6^  **Appropriate care:** It is likely a prevalence measure, which is less useful to quality improvement initiatives (e.g., to determine whether treatment/prevention strategies are effective).  **Clinical evidence**: This measure is based on appropriate evidence,^7^ although the grading system in use has not been evaluated recently.  **Specifications:** *Clarity:* Numerator not well defined and whether this is an incidence or prevalence measure is unclear. Each of the 6 stages of pressure injury are well defined. However, it is not specifically stated what the overall indicator refers to, i.e., not clear whether prevalence of residents with pressure injuries equates to at least one of the 6 categories. The denominator excludes individuals who withhold consent, which could result in selection bias. *Validity:* No information about validation of the measure is reported. *Reliability*: No information about accuracy and repeatability of measures (including inter-rater reliability) is provided; no estimates of precision are provided (e.g. confidence intervals). *Risk adjustment:* Measure is not risk adjusted, which is crucial for this outcome indicator (e.g., difference in mobility affects risk).  **Feasibility and applicability**: *Usability:* Measure cannot be used for benchmarking purposes (not risk adjusted) or associated with improved care (i.e., full skin inspections should occur more often than recommended). *Burden:* This varies across facilities due to assessment requirements (e.g., trained nursing staff, operational challenges). |
| **Physical restraints** | **Importance:** High impact event that needs national improvement.^8,9^ It is an important issue that is readily assessed. Aligns with principles of care, dignity and respect of people in aged care.^8,9^ Some evidence that national performance gap exists.^6^  **Appropriate care:** Certain types of physical restraints should be used in only exceptional circumstances, however whether this measure can promote stopping of the intervention is unknown. For example, alternative options need to be provided and depend on multiple factors (e.g., staff, training). Consideration needs to be placed on the risks to other residents and the impact of disruptive behaviours on their quality of life and potential risk of harm.  **Clinical evidence base:** There are multiple definitions of physical restraints in the literature.^10^ However, there is significant evidence that certain types of physical restraints are associated with poor psychological and physical outcomes, including death, and may not be effective in preventing serious injury for the resident.^11^ The 3-day period of review is not well justified and from the existing evidence base is unclear why this was chosen.  **Specifications**: *Clarity:* It is unclear whether this is an incidence or prevalence measure. More details regarding the nature of the restraint would be useful given the different types of constraint, e.g., using bedrails to prevent a fall; using locked doors to keep residents from existing the building versus potentially more harmful restraints such as seclusion or keeping someone restrained in a chair. *Validity:* No information about validation of the measure is reported. *Reliability:* As the survey period is preannounced it may result in responder bias. Challenges in definition make it unlikely to be a repeatable measure; no estimates of precision (e.g., confidence intervals) are provided. *Risk adjustment:* Measure is not risk adjusted.  **Feasibility and applicability:** *Providers control:* If physical restraints are initiated by family members, it is unlikely that aged care providers can address this measure. *Usability:* Measure cannot be used for benchmarking purposes (not risk adjusted). *Burden:* There are significant operational challenges with implementing data collection due to nine required observational periods and required documentation of observations. |
| **Unplanned weight loss – significant** | **Importance:** High impact event with high prevalence, morbidity, mortality, and major individual consequences**.^12-14^** Some evidence that national performance gap exists.^6^  **Appropriate care:** It is not clear how useful this measure will be to influence processes involved in resident nutritional care.  **Clinical evidence**: International literature agrees weight loss is an important measure of quality,^7^ however the % of weight loss deemed significant is not universally agreed upon.  **Specifications:** *Clarity:* ‘Unplanned’ lacks a consistent definition. Individuals who withhold consent or having missing weights recorded are excluded creating opportunity for selection bias. *Validity:* No information about validation of the measure is reported. *Reliability:* It is difficult to repeat these measures in the aged care setting, no estimates of precision are provided (e.g. confidence intervals). *Risk adjustment*: Measure is not risk adjusted, especially important if residents have conditions that may lead to weight loss.  **Feasibility and applicability**: *Usability:* Measures cannot be used for benchmarking purposes (not risk adjusted). *Burden:* There is high burden associated with weighting residents, from both a residents and providers’ perspective. |
| **Unplanned weight loss – consecutive** | **Importance** High impact event with high prevalence, morbidity, mortality, and major individual consequences**. ^12-14^** Some evidence that national performance gap exists.^6^  **Appropriate care:** It is not clear how useful this measure will influence processes involved in resident nutritional care.  **Clinical evidence:** International literature agrees weight loss is an important measure of quality,^7^ however consecutive weight loss and the period of three months for the assessment are not well justified.  **Specifications:** *Clarity:* Unplanned lacks a consistent definition. The denominator excludes individuals who withhold consent, which could result in selection bias. *Validity:* No information about validation of the measure is reported. *Reliability:* It is difficult to repeat these measures in the aged care setting, no estimates of precision are provided. *Risk adjustment*: Measure is not risk adjusted, especially important if residents have conditions that may lead to weight loss.  **Feasibility and applicability:** *Usability:* Measures cannot be used for benchmarking purposes (not risk adjusted). *Burden:* There is high burden associated with weighting residents, from both a resident’s and provider’s perspective. |
| **Falls -- one or more falls** | **Importance:** High impact event with high prevalence, morbidity, mortality, and major individual consequences**.^15-17^**  **Appropriate care:** The prevalence of falls is high. Unintended consequences associated with measuring falls include mobility restriction for older people.  **Clinical evidence**: There is international agreement that falls resulting in major injuries is an important measure of quality of care.^7^ While the factors contributing to falls are multiple some can be prevented.^16,18,19^ Unintended consequences associated with measuring falls include mobility restriction for older people.  **Specifications:** *Clarity:* Facility records are used to ascertain falls but language around falls’ assessment, which implies individual assessment is included. *Validity:* No information about validation of the measure is reported. *Reliability:* Measure relies on facility records and review of records, no information about accuracy and repeatability of measures (including inter and intra-rater reliability) is provided; no estimates of precision are provided (e.g. confidence intervals). *Risk adjustment*: Measure is not risk adjusted.  **Feasibility and applicability:** *Providers control*: Residents may accept higher risks of falls to maintain higher levels of function and mobility. *Usability:* Measures cannot be used for benchmarking purposes (not risk adjusted). *Burden:* Moderate burden with good facility documentation, however, it is unlikely all falls are recorded. |
| **Falls -- resulting in one or more major injuries** | **Importance:** High impact event with high prevalence, morbidity, mortality, and major individual consequences**.^15-17^**  **Appropriate care:** The prevalence of falls is high. Unintended consequences associated with measuring falls include mobility restriction for older people.  **Clinical evidence:** There is international agreement that falls resulting in major injuries is an important measure of quality.^7^ While the factors contributing to falls are multiple some can be prevented.^16,18,19^ Unintended consequences associated with measuring falls include mobility restriction for older people.  **Specifications:** *Clarity:* Facility records are used to ascertain falls with major injuries but language around falls’ assessment, which implies individual assessment, is included. It is unclear that discharge summaries from hospitals or other records needed to determine related major injuries would be readily available. Numerator: definition of major injuries can vary by assessor’s interpretation (e.g., did it require hospitalisation). *Validity:* No information about validation of the measure is reported. *Reliability:* Measure relies on facility records and review of records, no information about accuracy and repeatability of measures (including inter and intra-rater reliability) is provided, no estimates of precision are provided (e.g. confidence intervals). *Risk adjustment*: Measure is not risk adjusted.  **Feasibility and applicability:** *Providers control*: Residents may accept higher risks of falls to maintain higher levels of function and mobility. *Usability:* Measures cannot be used for benchmarking purposes (not risk adjusted). *Burden:* Moderate burden with good facility documentation, however it is unlikely all falls and related injuries are recorded. |
| **Medication management – polypharmacy** | **Importance:** Polypharmacy is highly prevalent and associated with a higher risk of morbidity, mortality, and major individual consequences.^20-23^  **Appropriate care:** The numerical definition of polypharmacy does not discriminate between appropriate and inappropriate medication use, therefore does not identify appropriate care and is not able to influence practice changes.  **Specifications:** *Clarity:* Identification of medications currently being administered is challenging. The denominator excludes individuals who are hospitalised on the day of assessment, which could result in selection bias especially given potential challenges associated with medication management that occur during transitions between hospital and residential care. *Validity:* No information about validation of the measure is reported. *Reliability:* Medication chart abstraction can be challenging and no information regarding inter and intra-rater reliability is reported. *Risk adjustment*: Measure is not risk adjusted.  **Feasibility and applicability:** *Providers control:* It is unlikely that the provider can influence the overall medication management of residents, this relies on medical practitioners managing their health conditions. *Burden:* Manual review of residents’ medication charts is very resource intensive. This would be feasible if facilities have electronic medication systems. *Usability:* This measure is not useful for improving care as it is not specific enough, it is also not risk adjusted and therefore does not allow for benchmarking comparisons. |
| **Medication management – antipsychotics** | **Importance:** Antipsychotics are used by more than 20% of residents in care yearly and are associated with significant morbidity, mortality, and major individual consequences.^24-29^ There is evidence that national performance gap exists.^24^  **Appropriate care:** There is significant evidence that inappropriate antipsychotic use in aged care is associated with poor outcomes and this measure could influence its use.  **Clinical evidence:** There is international agreement that antipsychotic use is an important measure of quality of care and that its use is associated with poor health outcomes for residents.^7^  **Specifications:** *Clarity:* An exhaustive list of antipsychotic medications is not included in the Manual Part A but only in the Manual Part B - this creates an opportunity for confusion. The denominator excludes individuals who are hospitalised on the week of assessment, which could result in selection bias. The denominator excludes individuals with a diagnosis of psychosis, which should be reported and monitored over time to ensure this does not increase artificially as a way of getting residents access to an antipsychotic. This measure should be stratified by the individuals’ dementia status as the main indication for these medications are for BPSD. Specification unclear regarding the appropriate/ legitimate use (short term) of risperidone for residents with Behavioural and Psychological Symptoms of Dementia  (BPSD). Duration of use is an important element not considered in this indicator. *Validity:* No information about validation of the measure is reported. *Risk adjustment*: Measure is not risk adjusted.  **Feasibility and applicability:** *Providers control:* While providers can contribute to managing individuals with dementia and BPSD, the prescribing of antipsychotics relies on medical practitioners managing their health conditions. *Burden*: Manual review of residents’ medication charts is very resource intensive. This would be feasible provided facilities have electronic medication systems. *Usability:* This measure is not risk adjusted or stratified by the dementia status of individuals which does not allow for meaningful benchmarking comparisons. |

**References**

1. Wilson L, Kapp S, Santamaria N. The direct cost of pressure injuries in an Australian residential aged care setting. Int Wound J 2019;16(1):64-70. DOI: 10.1111/iwj.12992.

2. Gorecki C, Brown JM, Nelson EA, et al. Impact of pressure ulcers on quality of life in older patients: a systematic review. J Am Geriatr Soc 2009;57(7):1175-83. DOI: 10.1111/j.1532-5415.2009.02307.x.

3. Lyder CH, Ayello EA. Pressure Ulcers: A Patient Safety Issue. In: Hughes RG, ed. Patient Safety and Quality: An Evidence-Based Handbook for Nurses. Rockville (MD)2008.

4. Song YP, Shen HW, Cai JY, Zha ML, Chen HL. The relationship between pressure injury complication and mortality risk of older patients in follow-up: A systematic review and meta-analysis. Int Wound J 2019;16(6):1533-1544. DOI: 10.1111/iwj.13243.

5. European Pressure Ulcer Advisory Panel and National Pressure Ulcer Advisory Panel (NPUAP). Prevention and Treatment of Pressure Ulcers/Injuries: Clinical Practice Guideline. 3rd edition. (<http://www.internationalguideline.com/static/pdfs/Quick_Reference_Guide-10Mar2019.pdf>).

6. Australian Government, Australian Insitute of Health and Welfare. Residential Aged Care Quality Indicators—Annual Report 2020-21. (<https://www.gen-agedcaredata.gov.au/www_aihwgen/media/2020-21-Quality-in-aged-care/RACS-Annual-Report-Quality-Indicators-2020-21.pdf>).

7. Caughey G, Lang C, Bray S, Wesselingh S, Inacio M. Research Paper 8 - International and National Quality and Safety Indicators for Aged Care. Report for the Royal Commission into Aged Care Quality and Safety. (<https://agedcare.royalcommission.gov.au/publications/research-paper-8-international-and-national-quality-and-safety-indicators-aged-care>).

8. Royal Commission into Aged Care Quality and Safety. Final report: Care, Dignity and Respect. (<https://agedcare.royalcommission.gov.au/sites/default/files/2021-03/final-report-volume-1_0.pdf>).

9. Royal Commission into Aged Care Quality and Safety. Interim Report: Neglect. (<https://agedcare.royalcommission.gov.au/publications/Documents/interim-report/interim-report-volume-1.pdf>).

10. Bleijlevens MH, Wagner LM, Capezuti E, Hamers JP, International Physical Restraint W. Physical Restraints: Consensus of a Research Definition Using a Modified Delphi Technique. J Am Geriatr Soc 2016;64(11):2307-2310. DOI: 10.1111/jgs.14435.

11. Hofmann H, Hahn S. Characteristics of nursing home residents and physical restraint: a systematic literature review. J Clin Nurs 2014;23(21-22):3012-24. DOI: 10.1111/jocn.12384.

12. Rasheed S, Woods RT. Malnutrition and quality of life in older people: a systematic review and meta-analysis. Ageing Res Rev 2013;12(2):561-6. DOI: 10.1016/j.arr.2012.11.003.

13. Banks M, Ash S, Bauer J, Gaskill D. Prevalence of malnutrition in adults in Queensland public hospitals and residential aged care facilities. Nutr Diet 2007;64(3):172-178. DOI: 10.1111/j.1747-0080.2007.00179.x.

14. Volkert D, Beck AM, Cederholm T, et al. Management of Malnutrition in Older Patients-Current Approaches, Evidence and Open Questions. J Clin Med 2019;8(7). DOI: 10.3390/jcm8070974.

15. Australian Government, Australian Institute of Health and Welfare. Trends in hospitalised injury due to falls in older people, 2007–08 to 2016–17. Injury research and statistics series no. 126. Cat. no. INJCAT 206. (<https://www.aihw.gov.au/getmedia/427d3a0d-88c2-45c5-bc23-5e3986375bba/aihw_injcat_206.pdf.aspx?inline=true>).

16. Inacio MC, Moldovan M, Whitehead C, et al. The risk of fall-related hospitalisations at entry into permanent residential aged care. BMC Geriatr 2021;21(1):686. DOI: 10.1186/s12877-021-02640-w.

17. World Health Organization. Global Report on Falls Prevention in Older Age. (<https://www.who.int/publications/i/item/who-global-report-on-falls-prevention-in-older-age?ua=1>).

18. Deandrea S, Bravi F, Turati F, et al. Risk factors for falls in older people in nursing homes and hospitals. A systematic review and meta-analysis. Arch Gerontol Geriatrics 2013;56(3):407-415. DOI: <https://doi.org/10.1016/j.archger.2012.12.006>.

19. Woolcott JC, Richardson KJ, Wiens MO, et al. Meta-analysis of the impact of 9 medication classes on falls in elderly persons. Arch Intern Med 2009;169(21):1952-1960. (In eng). DOI: 10.1001/archinternmed.2009.357.

20. Fried TR, O’Leary J, Towle V, Goldstein MK, Trentalange M, Martin DK. Health outcomes associated with polypharmacy in community-dwelling older adults: a systematic review. J Am Geriatr Soc 2014;62(12):2261–72.

21. Shah BM, JHajjar ER. Polypharmacy, adverse drug reactions, and geriatric syndromes. Clin Geriatric Med 2012;28(2):173-86.

22. Gutiérrez-Valencia M, Izquierdo M, Cesari M, Casas-Herrero Á, Inzitari M, Martínez-Velilla N. The relationship between frailty and polypharmacy in older people: A systematic review. Br J Clin Pharmacol 2018;84(7):1432-1444. (In eng). DOI: 10.1111/bcp.13590.

23. Storms H, Marquet K, Aertgeerts B, Claes N. Prevalence of inappropriate medication use in residential long-term care facilities for the elderly: a systematic review. Eur J Gen Pract 2017;23(1):69-77.

24. Inacio MC, Lang C, Caughey GE, et al. The Registry of Senior Australians outcome monitoring system: quality and safety indicators for residential aged care. Int J Qual Health Care 2020;32(8):502-510. DOI: 10.1093/intqhc/mzaa078.

25. Harrison SL, Sluggett JK, Lang C, et al. The dispensing of psychotropic medicines to older people before and after they enter residential aged care. Med J Aust 2020;212(7):309-313. DOI: 10.5694/mja2.50501.

26. Kirkham J, Sherman C, Velkers C, et al. Antipsychotic Use in Dementia. Can J Psych 2017;62(3):170-181. (In eng).

27. Ballard C, Hanney ML, Theodoulou M, et al. The dementia antipsychotic withdrawal trial (DART-AD): long-term follow-up of a randomised placebo-controlled trial. The Lancet Neurology 2009;8(2):151-7. (In eng). DOI: 10.1016/s1474-4422(08)70295-3.

28. Rochon PA, Stukel TA, Sykora K, et al. Atypical Antipsychotics and Parkinsonism. JAMA Internal Medicine 2005;165(16):1882-1888. DOI: 10.1001/archinte.165.16.1882.

29. Vigen CLP, Mack WJ, Keefe RSE, et al. Cognitive effects of atypical antipsychotic medications in patients with Alzheimer's disease: outcomes from CATIE-AD. Am J Psych 2011;168(8):831-839. (In eng). DOI: 10.1176/appi.ajp.2011.08121844.
